# Advanced endoscopic rescue after failed endoscopic ultrasound–guided choledochoduodenostomy

**DOI:** 10.1016/j.vgie.2026.02.015

**Published:** 2026-03-12

**Authors:** Stefan Michaelis, Ulrich Rosien, Alexander Arlt

**Affiliations:** Department of Internal Medicine and Gastroenterology, Israelitisches Krankenhaus, Hamburg, Germany

## Abstract

**Background and Aims:**

EUS-guided choledochoduodenostomy (EUS-CDS) has become an established alternative to percutaneous or surgical approaches in cases of failed or predicted difficult ERCP.

**Methods:**

We report a case of complex endoscopic management of biliary obstruction in a 46-year-old woman with inoperable pancreatic adenocarcinoma. Because of tumor-related deformation of the papillary region, primary transpapillary drainage was not feasible.

**Results:**

EUS-CDS using a 6- × 8-mm electrocautery-enhanced lumen-apposing metal stent (LAMS) (Hot AXIOS stent; Boston Scientific, Marlborough, Mass, USA) was performed. Misdeployment of the distal flange led to bile leakage and stent dysfunction. Subsequent placement of a fully covered self-expandable metal stent (8 mm × 6 cm) (BONASTENT; MTW, Wesel, Germany) resulted in secondary stent dislocation into the paraduodenal space. The dislocated stent was successfully retrieved endoscopically through the original access using a 3-mm cholangioscope (Scivita, Boston Scientific). The duodenal defect was closed with 2 closure clips (MANTIS Clip, Boston Scientific). The patient recovered uneventfully and remained asymptomatic at 2-month follow-up.

**Conclusions:**

This case illustrates the feasibility of endoscopic rescue in a challenging scenario after LAMS misdeployment using advanced ERCP and closure techniques.

## Introduction

EUS-guided biliary drainage (BD) has become an established alternative to percutaneous or surgical approaches in cases of failed or predicted difficult ERCP.^1^^,^^2^ Among the various techniques, EUS-guided choledochoduodenostomy (EUS-CDS) using lumen-apposing metal stents (LAMSs) allows for direct internal BD. However, stent misdeployment or migration remains a rare but severe adverse event, potentially leading to bile leakage, peritonitis, or duodenal perforation.^3^

**Figure 1:**
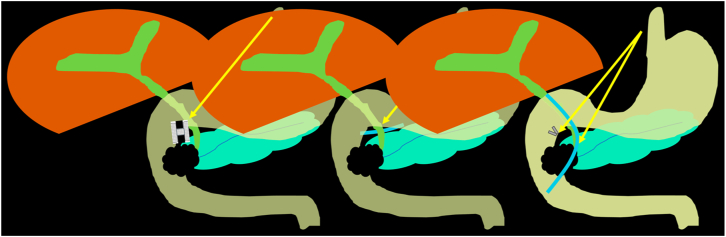
Cartoon image of the dislocated stents and the final biliary fully covered self-expandable metal stent. *Left arrow* indicates LAMS outside of the CBD. *Middle arrow* indicates SEMS in the paraduodenal cavity. *Right arrows* indicate clips in the duodenum and SEMS in the CBD. *CBD*, Common bile duct; *LAMS*, lumen-apposing metal; *SEM*, self-expandable metal stent.

We present a case of endoscopic rescue after misdeployment of a Hot AXIOS (Boston Scientific, Marlborough, Mass, USA) stent with subsequent biliary and duodenal adverse events (Fig. 1).

## Case presentation

**Figure 2:**
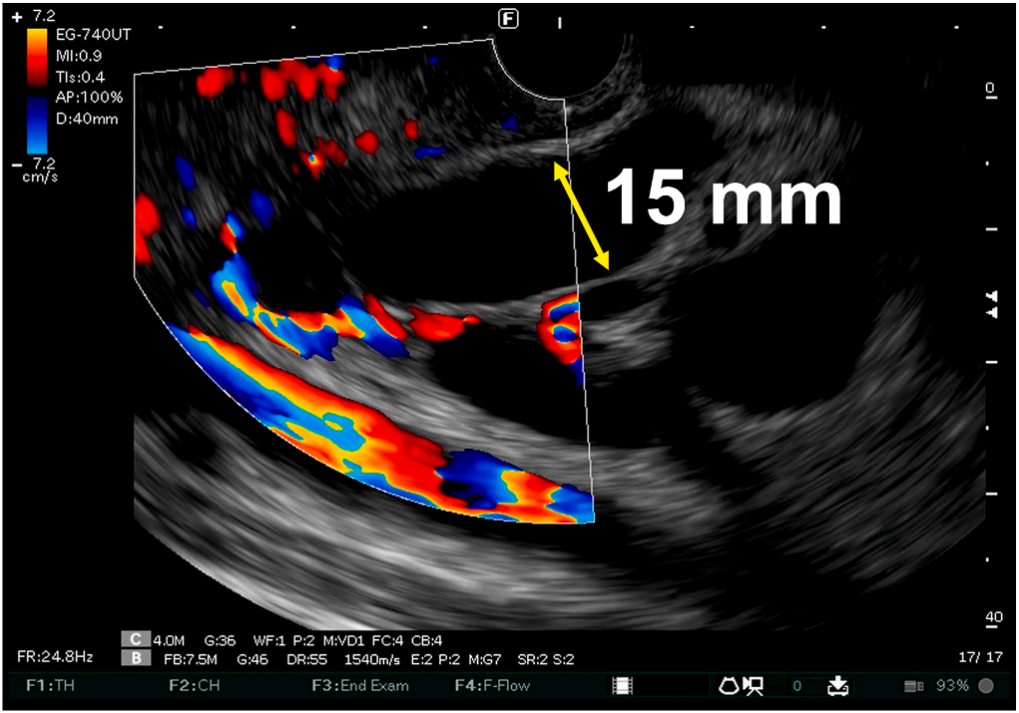
EUS showing the dilated common bile duct before lumen-apposing metal stent placement. *Arrow* shows the diameter of the common bile duct.

A 46-year-old woman with an inoperable pancreatic head carcinoma presented with progressive jaundice and pruritus because of malignant biliary obstruction. Cross-sectional imaging revealed tumor infiltration and compression of the distal bile duct as well as deformation of the papillary region. Because of the anatomical distortion, ERCP access to the papilla was very difficult. In the EUS, the bile duct measured 15 mm from the duodenal bulb (Fig. 2)

**Figure 3:**
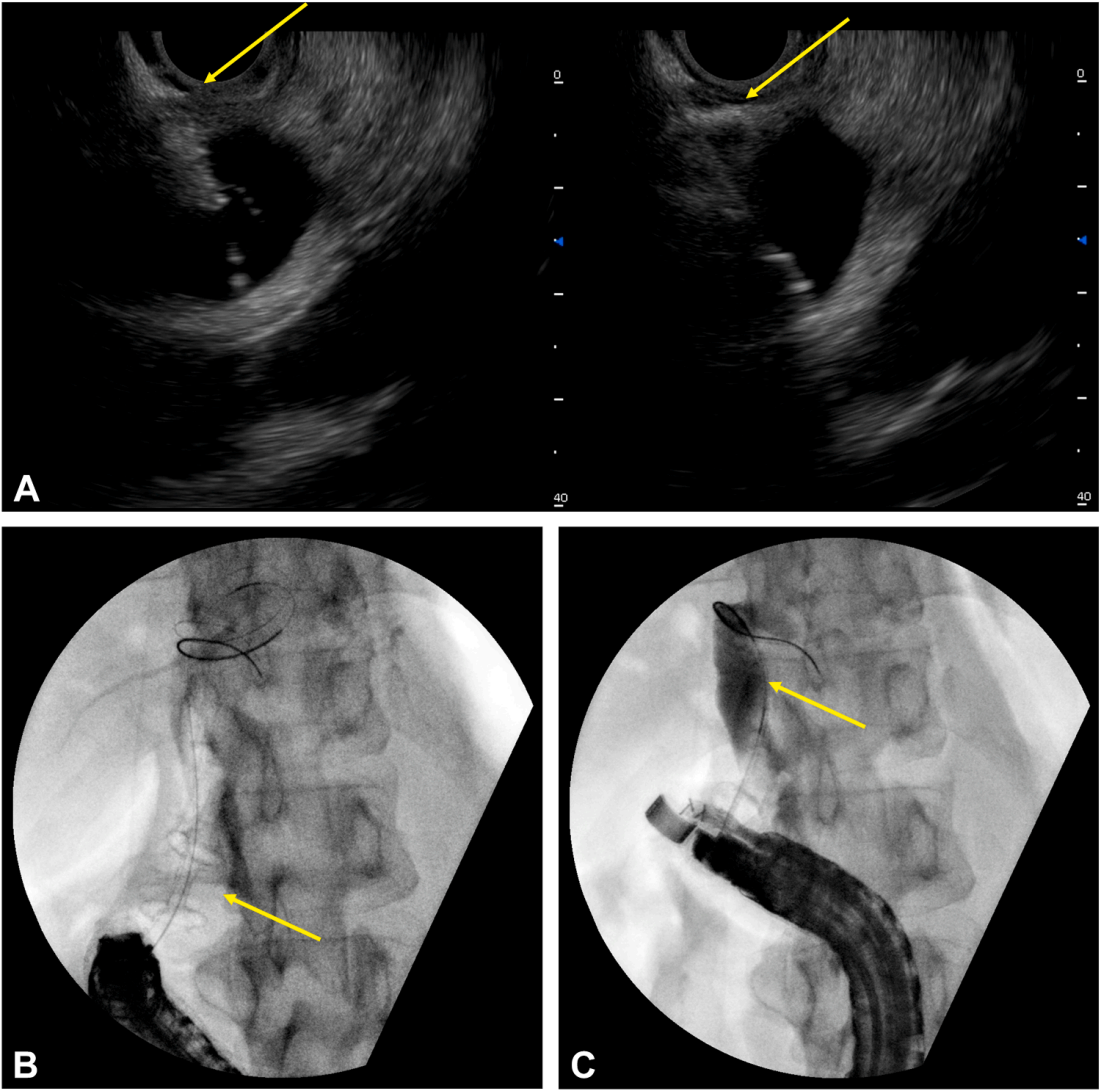
**A**, EUS image demonstrating misdeployment of the distal flange of the Hot AXIOS lumen-apposing metal stent (LAMS) (Boston Scientific, Marlborough, Mass, USA) with incomplete bile duct apposition. *Arrow* indicates the distal flange of the Hot AXIOS LAMS. **B**, Fluoroscopic image demonstrating misdeployment of the distal flange of the Hot AXIOS LAMS with incomplete bile duct apposition. *Arrow* indicates the middle of the Hot AXIOS LAMS. **C**, Fluoroscopic image demonstrating the wire through the LAMS in the intrahepatic bile duct system. *Arrow* indicates wire in the bile duct.

After multidisciplinary discussion, EUS-CDS was performed using a 6- × 8-mm Hot AXIOS stent. During deployment (Video 1, available online at www.videogie.org), the distal flange was misdeployed, resulting in an incomplete apposition of the bile duct wall and no effective BD (Fig. 3A and B).

**Figure 4:**
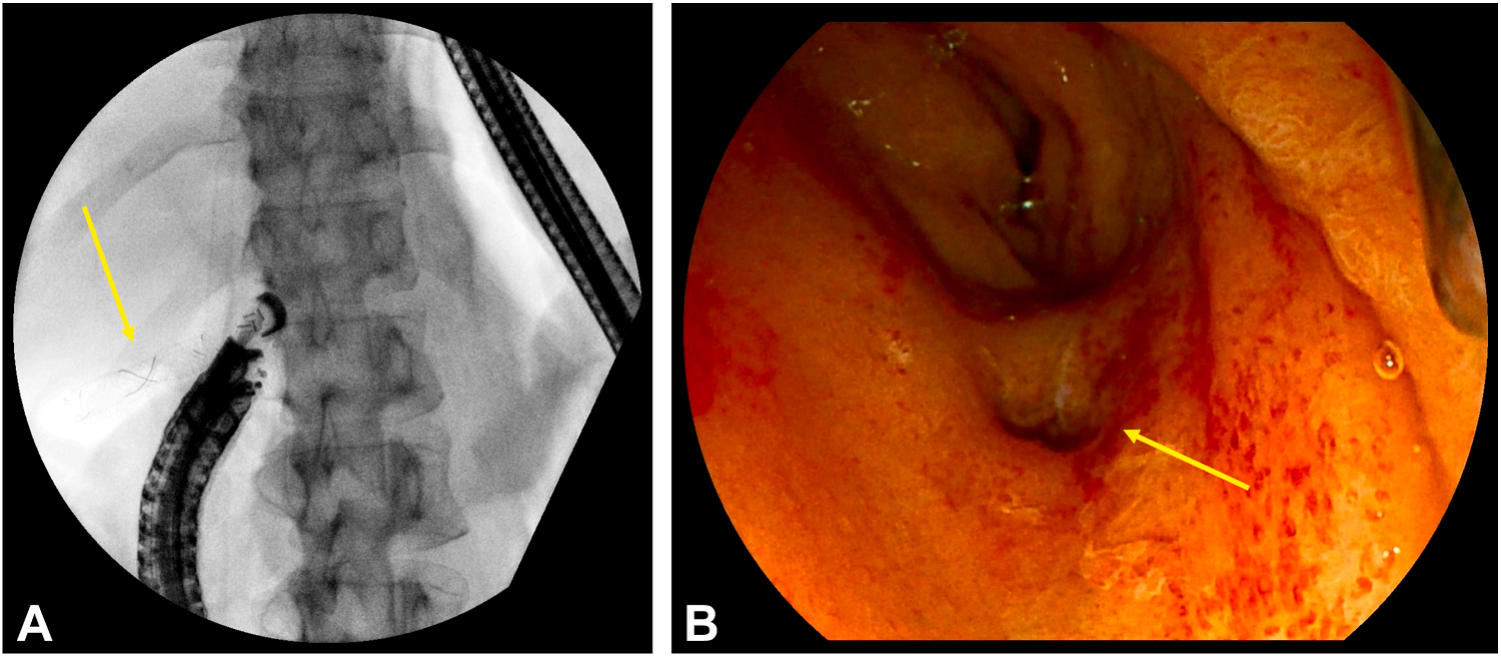
**A**, Fluoroscopic image showing dislocation of the fully covered self-expandable metal stent into the preduodenal space. *Arrow* indicates the FCSEMS. **B**, Endoscopic image of the duodenal opening without the dislocated stent. *Arrow* indicates the opening in the duodenal wall.

Under EUS and fluoroscopic guidance, a guidewire was advanced through the misplaced stent into the intrahepatic biliary system (Fig. 3C). The LAMS was removed, and a fully covered self-expandable metal stent (FCSEMS, 8 mm × 6 cm, BONASTENT; MTW, Wesel, Germany) was placed across the choledochoduodenal fistula. Under endoscopic and fluoroscopic control, the stent was initially correctly positioned. Directly after the release, the stent started to dislocate initially from the bile duct and then from the duodenum into the paraduodenal space, resulting in complete extraluminal dislocation and a perforation defect in the bile duct and duodenum (Fig. 4).

## Rescue procedure

**Figure 5:**
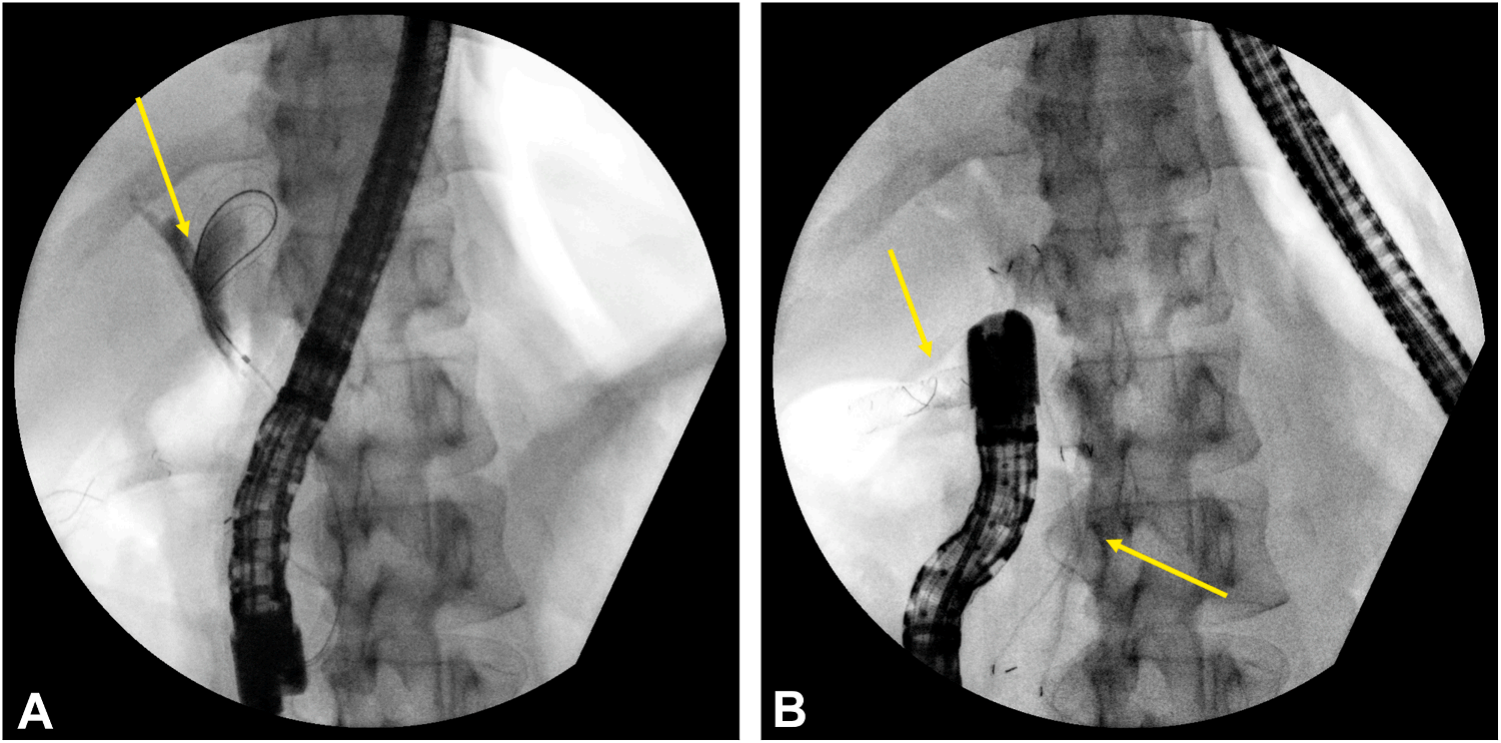
**A**, Fluoroscopic image demonstrating the wire (*arrow*) positioned by endoscopic retrograde cholangiography in the intrahepatic bile duct system. **B**, Fluoroscopic image after transpapillary placement of a 10- × 8-cm fully covered metal stent restoring biliary drainage and the dislocated initial fully covered self-expandable metal stent in the paraduodenal space. *Left arrow* indicates dislocated initial FCSEMS and *right arrow* indicates the correctly positioned FCSEMS in the common bile duct.

The patient remained stable without a relevant pneumoperitoneum, and, in agreement with the surgical department, an endoscopic approach to address these adverse effects was chosen. To close the leakage of the bile duct, a second attempt at BD was performed by ERCP. The papilla was reached with the duodenoscope after a forced dilatation maneuver of the duodenal lumen. After difficult wire-guided cannulation of the common bile duct (CBD), a sphincterotomy was performed, and a 10-mm × 8-cm FCSEMS (WallFlex, Boston Scientific) was successfully inserted transpapillarily by standard endoscopic retrograde cholangiography, sealing the hole in the CBD and restoring BD (Fig. 5).

**Figure 6:**
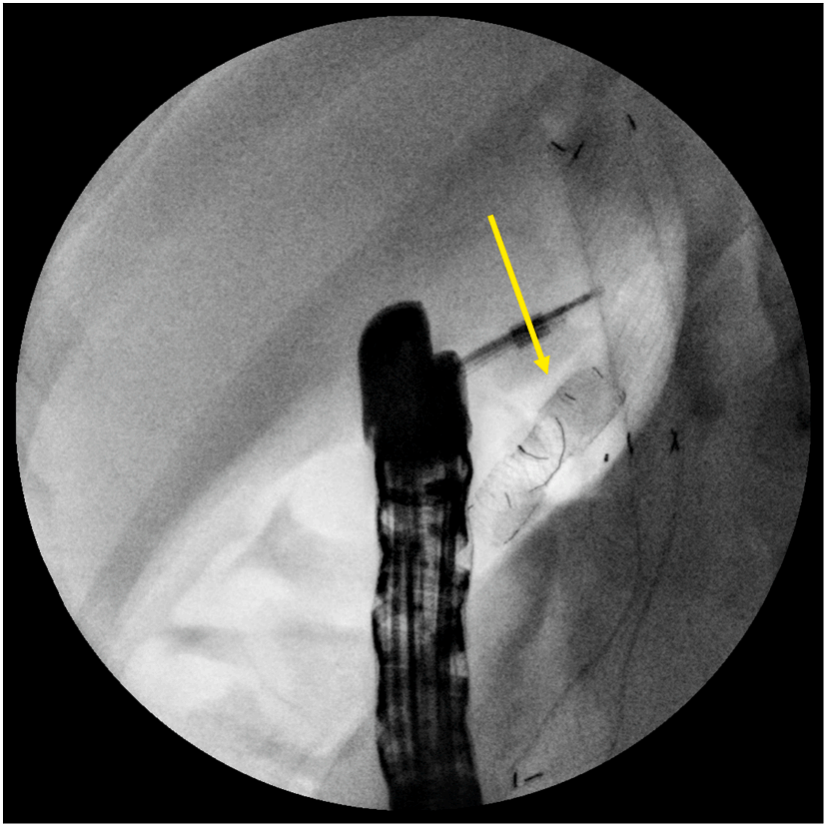
Fluoroscopic image demonstrating the failed retrieval of the dislocated fully covered self-expandable metal stent through the defect of the duodenal wall through a duodenoscope. *Arrow* indicates dislocated initial FCSEMS.

Efforts to retrieve the migrated stent through the duodenal defect were initially unsuccessful (Fig. 6). Because the defect was still over 5 mm in diameter, a 3-mm digital cholangioscope (Scivita, Boston Scientific) was advanced through a duodenoscope and the duodenal wall defect into the paraduodenal cavity. Under direct visualization, the dislocated stent was identified and retrieved endoscopically using cholangioscopy biopsy forceps (SpyBite Max, Boston Scientific) (Video 2, available online at www.videogie.org).

**Figure 7:**
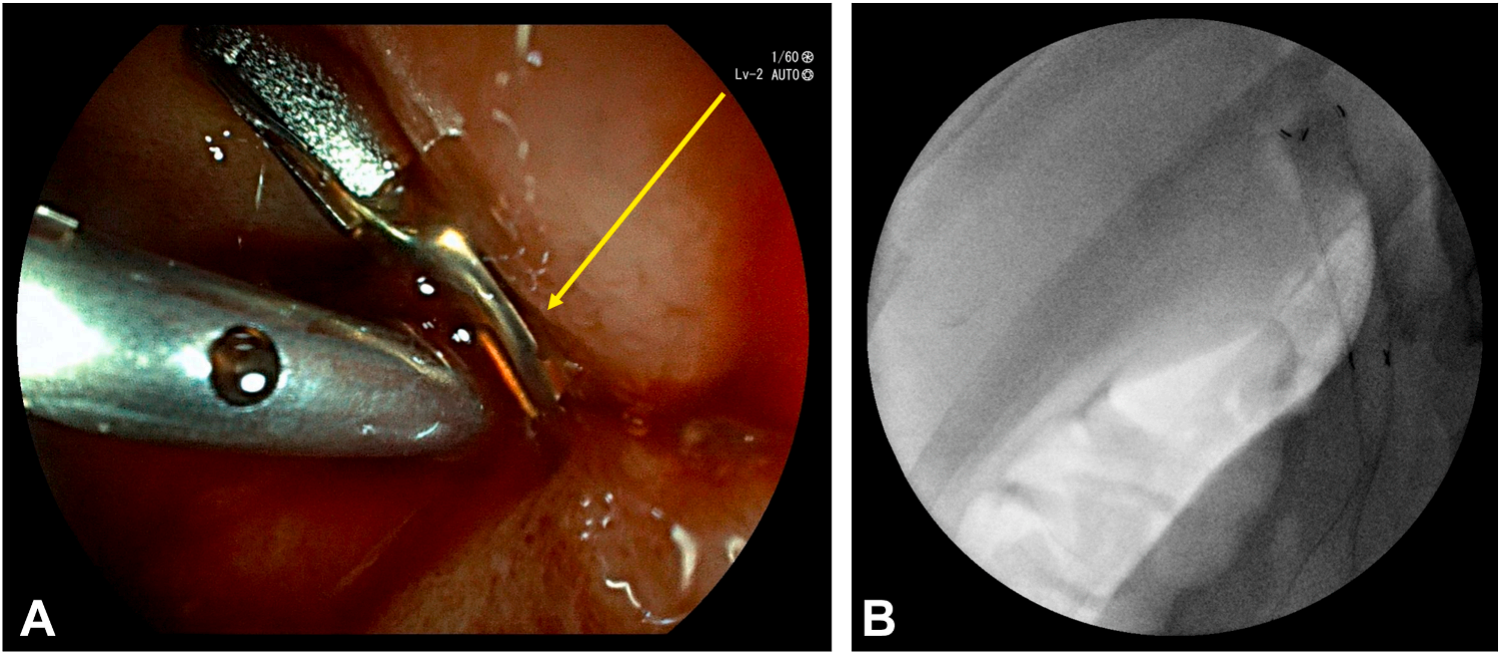
**A**, Endoscopic image showing complete closure of the duodenal defect with 2 closure clips. *Arrow* indicates the clips. **B**, Fluoroscopic image demonstrating successful drainage with a biliary fully covered self-expandable metal stent and closure of the duodenal wall.

After complete removal, the duodenal defect was closed using 2 closure clips (MANTIS Clip, Boston Scientific), achieving complete closure under endoscopic control (Fig. 7). Through-the-scope clips were chosen over an over-the-scope clip to avoid an accidental closing of the bile duct lying directly behind the defect of the duodenal wall.

## Postprocedural management

**Figure 8:**
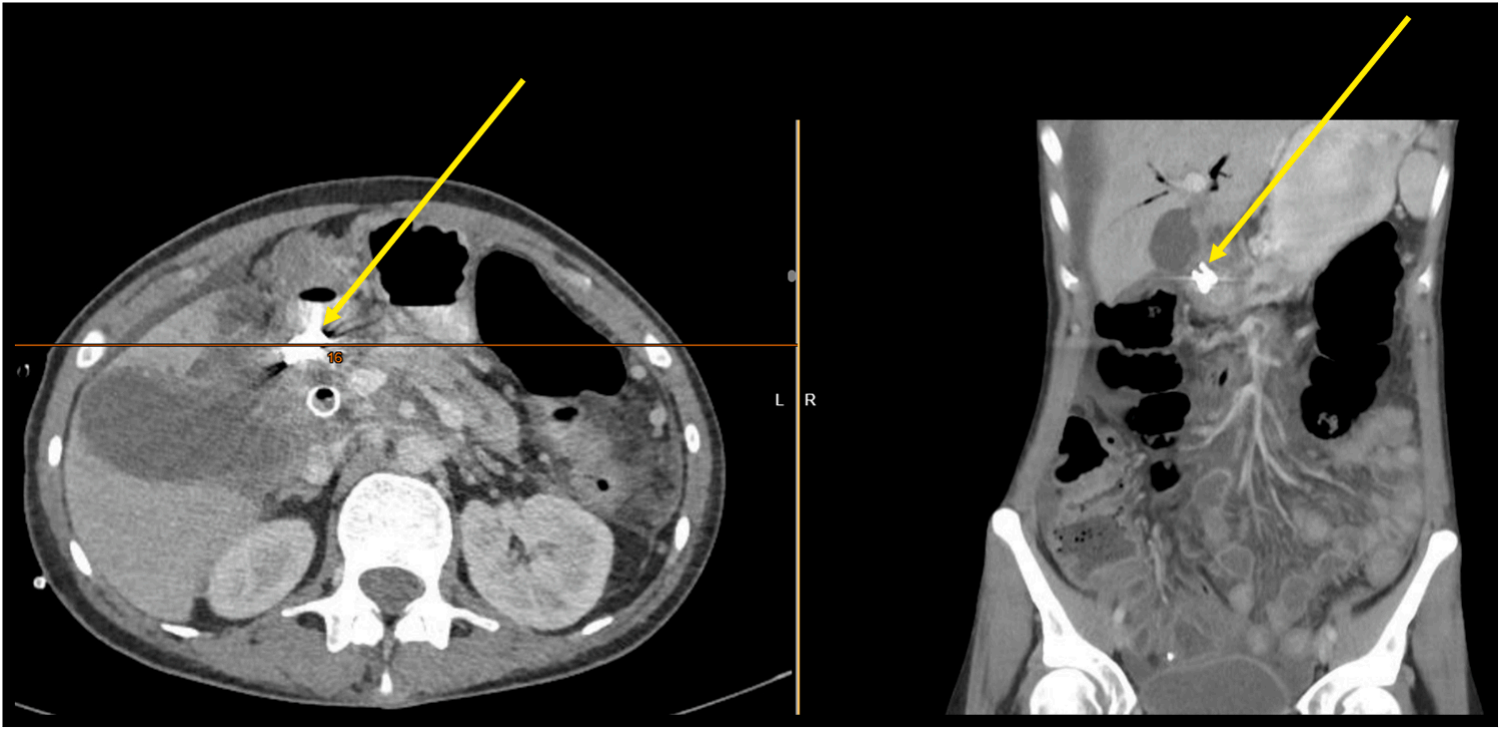
CT scan with oral contrast confirming sufficient closure of the duodenal wall. *Arrow* indicates the clips.

The complete procedure lasted 90 minutes and was performed using CO_2_ for insufflation. The patient received a single dose of a broad-spectrum cephalosporin and was monitored in the intensive care unit for 12 hours. CT scan with oral contrast confirmed successful closure of the duodenal wall (Fig. 8). Oral feeding was reintroduced 24 hours later. The postoperative course was uneventful, and the patient was discharged 4 days after the procedure in good condition.

## Follow-up

At the 2-month follow-up, the patient remained asymptomatic with normal bilirubin levels. Cross-sectional imaging confirmed stable positioning and function of the biliary stent, without signs of leakage, infection, or abscess formation. The underlying pancreatic malignancy remained unresectable and was managed palliatively.

## Discussion

EUS-guided BD has emerged as a key technique for patients in whom ERCP is not feasible.^1^^,^^2^ LAMS devices, such as Hot AXIOS, simplify the procedure but carry specific risks of maldeployment, migration, and bile leak.^3^

In this case, the distal flange misdeployment led to an incomplete choledochoduodenostomy, followed by direct dislocation of a rescue FCSEMS. Such a combination of adverse effects is exceedingly rare and potentially life-threatening.

Our management strategy included (1) guidewire salvage through the maldeployed stent to maintain biliary access; (2) secondary transpapillary stent placement to re-establish drainage; (3) cholangioscopic retrieval of the migrated stent under direct vision; and (4) endoscopic closure of the duodenal wall defect using advanced clip technology.

This multistep approach avoided surgery in a patient with advanced malignancy and poor performance status. The use of a 3-mm cholangioscope proved crucial for visualization and safe removal of the displaced stent.

## Conclusion

Endoscopic rescue after LAMS misdeployment and stent migration is feasible in expert hands. Maintaining guidewire access, using advanced endoscopic procedures outside the GI lumen, and using advanced closure techniques are essential components of a successful minimally invasive management strategy in such catastrophic scenarios.

## Patient consent

The patient in this article has given written informed consent to publication of her case details.

## Ethical statement

All procedures were performed in accordance with institutional and ethical standards and the Declaration of Helsinki.

## Disclosure

The following author disclosed financial relationships: A. Arlt: Consultant for Boston Scientific, Fujifilm, and Olympus. All other authors disclosed no financial relationships.

## Supplementary data

Videos Video 1. EUS-guided choledochoduodenostomy with a 6 × 8-mm lumen-apposing metal stent until release of the distal flange. Video 2. Real-time cholangioscopic visualization of the migrated stent in the paraduodenal cavity and its retrieval using a 9F digital cholangioscope and a cholangioscopy biopsy forceps.
